# Long-Wavelength Beam Steerer Based on a Micro-Electromechanical Mirror

**DOI:** 10.6028/jres.118.006

**Published:** 2013-03-01

**Authors:** Anthony B Kos, Eyal Gerecht

**Affiliations:** National Institute of Standards and Technology, Boulder, CO 80305

**Keywords:** DSP, image raster-scanning, long-wavelength, long-wavelength beams, long-wavelength imaging, MEMS, quasi-optical coupling, terahertz, terahertz beams, terahertz imaging, terahertz switching elements

## Abstract

Commercially available mirrors for scanning long-wavelength beams are too large for high-speed imaging. There is a need for a smaller, more agile pointing apparatus to provide images in seconds, not minutes or hours. A fast long-wavelength beam steerer uses a commercial micro-electro-mechanical system (MEMS) mirror controlled by a high-performance digital signal processor (DSP). The DSP allows high-speed raster scanning of the incident radiation, which is focused to a small waist onto the 9mm^2^, gold-coated, MEMS mirror surface, while simultaneously acquiring an undistorted, high spatial-resolution image of an object. The beam steerer hardware, software and performance are described. The system can also serve as a miniaturized, high-performance long-wavelength beam chopper for lock-in detection.

## 1. Introduction

Spectroscopy and imaging at long wavelengths have great potential for a wide range of healthcare and remote sensing applications. Radiation at terahertz frequencies corresponds to unique vibrational and rotational energy level transitions of biochemical molecules. This radiation also has a shorter wavelength than the microwave/millimeter wave range, allowing imaging with sub-millimeter spatial resolution.

We have developed quasi-optical terahertz systems based on heterodyne detection [1,2]. Traditional quasi-optical system configurations in the terahertz range incorporate a combination of a dielectric lens and an integrated monolithic antenna to couple the terahertz beams to the detectors. Gold coated mirrors are used to reflect and focus terahertz beams. Recent advances have produced terahertz imagers based on beam steering in order to image an object [1]. Both active detection, in which the object is illuminated to improve sensitivity, and passive detection schemes have been demonstrated. Images with a large number of pixels can be produced with either a large focal plane array (FPA) with many detectors (and the additional complexity of multiplexing the signals from all the detectors to render an image), or a single detector to scan the imaging beam across the subject. Commercial scanning mirrors are relatively large and slow. There is a need for small steering mirrors that can scan long-wavelength beams very fast (line scans in seconds). The small *waist* of a focused terahertz beam, only a few hundreds of micro-meters wide, can be steered by a gold coated mirror with a physical dimension of only a few millimeters.

Moreover, a general type of configuration used for microwave or terahertz radiometers involves switching the input source in order to eliminate the effect of system gain fluctuations [3]. One common technique to implement such switching is known as the Dicke receiver configuration [4], where the receiver collects the radiation signal and compares it with a known reference, usually a blackbody source with a well-controlled temperature, at a rate given by the switching frequency *f*_m_. A lock-in amplifier is then utilized to detect and integrate the output signal for improved sensitivity while reducing the system susceptibility to gain fluctuations at the expense of scanning speed. We have demonstrated imaging systems with switching frequencies of up to 200 Hz using a commercially available mechanical chopper [1]. Faster switching frequencies, a few kilohertz, are desired in order to improve the overall sensitivity and speed of terahertz imaging systems based on the Dicke configuration.

In 1987, the digital micro-mirror device (DMD) was invented [5] for light projection. The chip consists of several hundred thousand microscopic mirrors and was the predecessor of the digital light processing (DLP) [6] technology that is the core of widely available projection televisions. Each mirror can be controlled separately and represents a pixel in the image. The mirrors are fabricated in a focal plane array configuration. The DMD is based on micro-electromechanical system (MEMS) technology used in a number of applications requiring switching electronics. Fast optical switching circuits are routinely used in telecommunication networks using fiber optics.

Taking advantage of the relatively short wavelengths of terahertz radiation and the advent of MEMS devices in terms of their physical size and speed, we have developed a new system to steer these long-wavelength beams. We termed the system MEMS Long-wavelength Beam Steerer (MLBS).

## 2. Design and Architecture of MLBS System

For high-speed imaging we need to rapidly scan the incident radiation across the imaged sample without distorting the shape of the beam. Depending on the size of the sample, the apparatus is required to scan over a range of millimeters to centimeters at the object plane. Images can range from a small number of pixels (a few hundred) for high speed acquisition to a large number of pixels (many thousands) at the expense of speed. The beam shape and position control have to be very high in order to produce undistorted high-spatial resolution images. The pixel scan rate must be controlled by the user and can be as slow as 100 ms per pixel or as fast as 0.1 ms per pixel in order to accommodate the signal integration time of the detector.

**Fig. 1:**
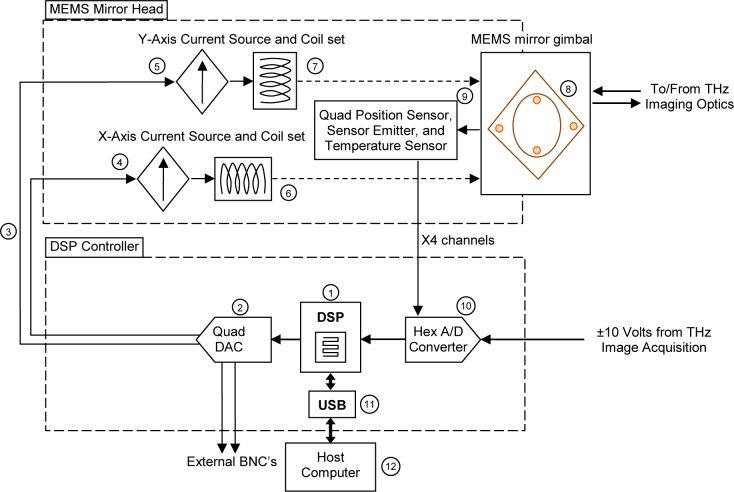
Block diagram of the MLBS showing MEMS Mirror Head and DSP Controller. Shown in the figure are the DSP with USB interface, 6-channel ADC, Quad DAC, Dual Current Source, and Dual Axis MEMS Analog Mirror.

We designed, fabricated, and analyzed a MEMS-based steering mirror system to achieve these scanning beam requirements. Our MLBS system is based on a MEMS chip [7] provided to us by a group at Texas Instruments (TI) working on the DLP technology used for visible light projection. Other sources of commercial MEMS mirror chips are also available [8]. Instead of using an array of MEMS mirrors that propagates or blocks (via digital logic) a beam for each pixel, we used a single mirror that can be steered ±5 degrees very rapidly and reproducibly. This technique has been used successfully for optical imaging [9]. Figure 1 shows the block diagram of the MLBS. The basic operation is as follows: the digital signal processor (DSP) (1) outputs the x and y values of each pixel position to a quad channel, 16-bit digital to analog converter (DAC) (2) for conversion into ±10 volts. This x and y voltage signal (3) is applied to the inputs of two voltage-programmable bipolar current sources (4) and (5), which supply up to ±20 mA of drive current each to two sets of mirror positioning coils (6) and (7) integrated into the mirror assembly. The mirror positioning coil sets push or pull on two small pairs of magnets mounted on the mirror and gimbal assembly (8), which sets the position of the mirror in two axes. Four optical position sensors integrated into the mirror/gimbal assembly (9) are used to provide 13-bit closed-loop position feedback as well as faster mirror switch times. After the mirror positioning is completed, the image acquisition signal is digitized by a 6-channel analog to digital converter (ADC) (10). In addition, this ADC also acquires the position feedback signals from the four optical sensors for closed-loop mirror positioning. The image acquisition values from the ADC are sent to the DSP (1) for storage as a single line scan. After a line scan is completed the data are sent over a high-speed universal serial bus (USB) interface (11) to the host computer (12). The host computer can also send commands to the DSP to control all aspects of scanning and data acquisition.

The MLBS system consists of two major hardware components: the MEMS mirror head, and the DSP controller. The system software for control consists of: the firmware that resides inside the DSP controller, and the host program, or graphical user interface (GUI), that runs on the host computer.

### 2.1 Hardware Components

#### 2.1.1 MEMS Mirror Head

**Fig. 2:**
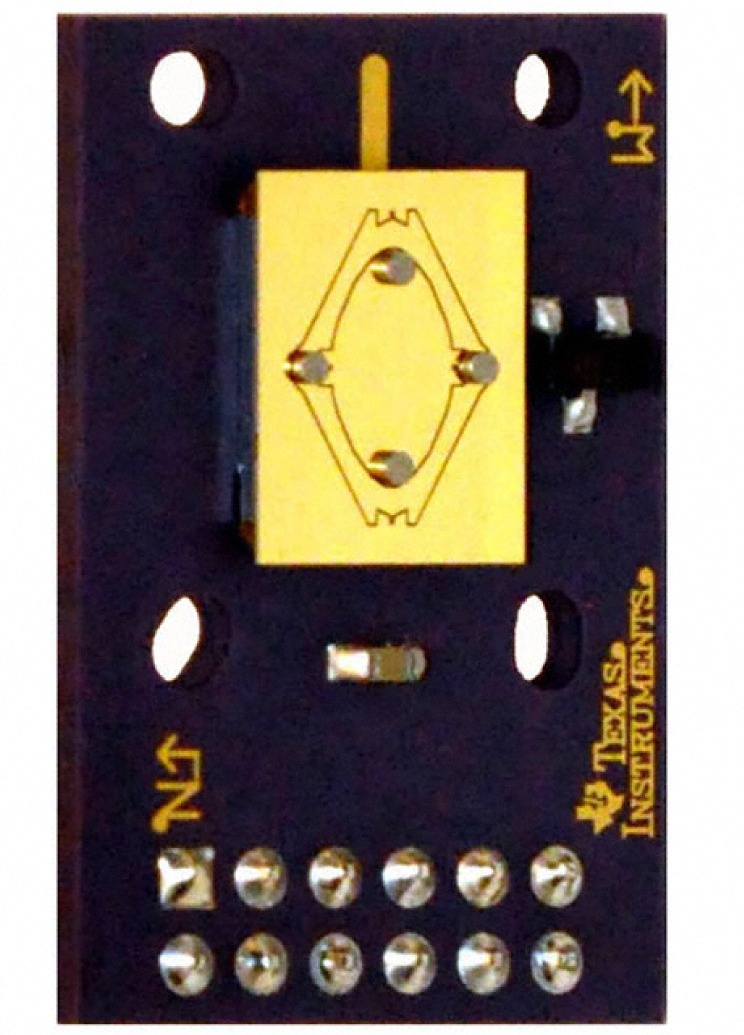
Photograph of the MEMS Analog Mirror showing two axis gimbaled mirror surface, both pairs of actuating magnets, manufacturer’s directional marks, mounting holes, and 12-pin connector interface.

**Fig. 3:**
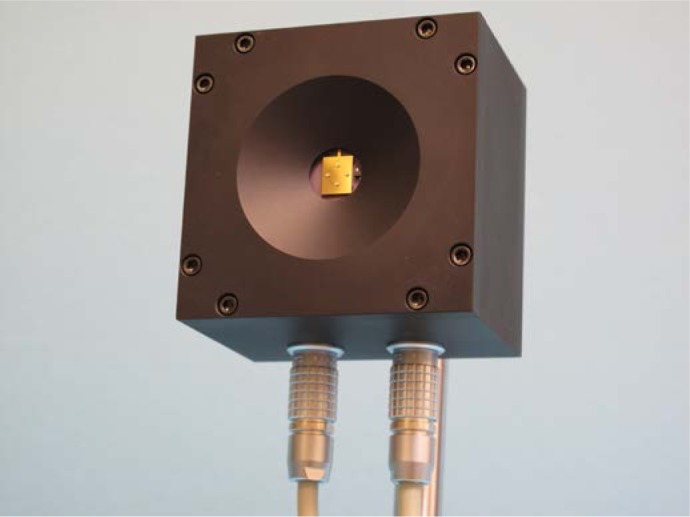
Photograph of the MEMS Mirror Head assembly showing mirror surface, custom aluminum enclosure, analog input/output and power connectors, and mounting hardware.

The MEMS-based mirror is a TI TALP1000B^1^ Dual-Axis Analog MEMS Pointing Mirror [7] shown in Fig. 2. Figure 3 shows the MEMS mirror assembled in a custom-machined aluminum enclosure along with its external connectors. This mirror is made of single crystal silicon with no grain boundaries and is coated with gold to create the mirror surface. The specified wavelength range over which suitable reflectivity occurs is 700 nm to 10 µm (mid-infrared). We used the mirror in the far-infrared (350 µm), since in this regime the MEMS gold mirror, with surface roughness of 200 nm, is an even more suitable reflector, relative to the wavelength. The mirror is over 9 mm^2^ in size, with a radius of curvature specified to be greater than 5 m.

Mirror actuation is provided via an electromagnetic drive, consisting of coils and small permanent magnets arranged to yield up to ±5 degrees of mirror rotation in both axes. Each axis has its own drive and can be independently actuated, with crosstalk between the two axes specified to be no greater than 5 %. The current necessary to drive the coils is provided by two independent operational amplifier current sources capable of supplying up to ±20 mA of drive current, programmed by two ±10 V DAC channels from the DSP controller.

The TALP1000B comes with integrated optical position feedback which is specified at greater than 13-bits of pointing precision. The four signals comprising this position feedback are sent to the DSP controller where they are digitized with a 16-bit ADC. These four signals are designated by TI as NW, SW, SE, and NE, i.e., making use of the four ordinal directions (or points of the compass). This nomenclature is confusing for our application and so we rename them to X^+^Y^+^, X^−^Y^+^, X^−^Y^−^ and X^+^Y^−^, which is the direction they would indicate if one stands behind the mirror enclosure, with X^+^ on the right and Y^+^ pointing upward.

These four detector output signals indicate the size of the upper right, upper left, lower left, and lower right components of the position of the X and Y axes, which requires a computation to yield the actual X and Y axis positions:

$$Y=\frac{X^{+}Y^{-}+X^{-}Y^{-}-X^{+}Y^{+}-X^{-}Y^{+}}{X^{+}Y^{-}+X^{-}Y^{-}+X^{+}Y^{+}+X^{-}Y^{+}}$$ (1a)

$$X=\frac{X^{+}Y^{-}-X^{-}Y^{-}+X^{+}Y^{+}-X^{-}Y^{+}}{X^{+}Y^{-}+X^{-}Y^{-}+X^{+}Y^{+}+X^{-}Y^{+}}$$ (1b)

These four signals must be measured and X and Y positions re-calculated by the DSP controller for every position feedback iteration.

This mirror has several advantages over other mirrors seen in the literature. It has an extremely linear electromagnetic drive with no flat spots around zero, such as found in thermal actuators. It does not depend on resonance for operation and can be used for accurate static positioning. The large 9 mm^2^ mirror surface is well suited for long-wavelength imaging and convenient setup of optical experiments. The low voltage operation is an additional benefit of this mirror, unlike electrostatic mirrors which require voltages up to and above 100 volts. Drive waveforms are simple, linearly-swept, bipolar triangle voltage waveforms, since the mirror has a simple 2-axis gimbal without complicated hinging. The mirror has good imaging performance, operating to 20 scan lines per second below resonance and above resonance at 200 scan lines per second. Finally, the mirror does not have out-of-plane motion, since it is magnetically driven in a balanced electromagnetic push-pull configuration.

#### 2.1.2 DSP Controller

**Fig. 4:**
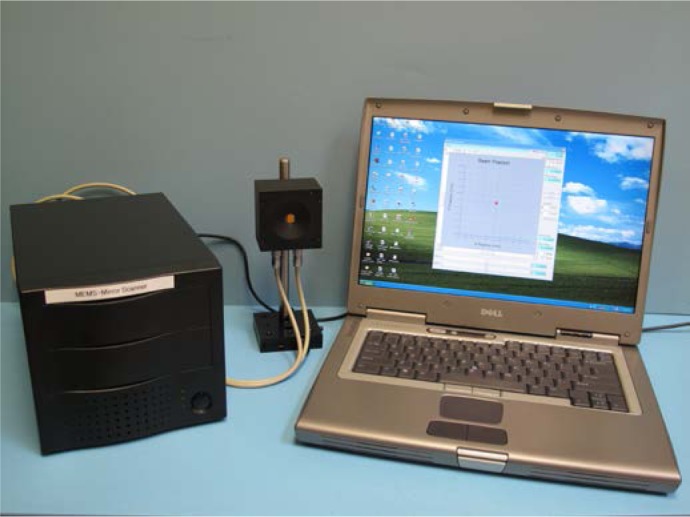
Picture of MLBS showing DSP controller, MEMS Mirror Head, laptop computer and interconnecting cables. Also shown is the GUI program running on the laptop.

The assembled system, showing the DSP controller, MEMS mirror head, mirror head cabling, USB cable and laptop computer, is shown in Fig. 4. The DSP controller box houses 4 main component boards: the DSP Starter Kit (DSK) board [10], the USB and quad-channel DAC board (USB/QDAC daughtercard), the analog to digital converter board (ADC daughtercard), and the power supply board. The TI TMS320C6416 DSP Starter Kit board features a 1 GHz C6416 32-bit fixed-point digital signal processor. Several important features of the C6416 DSK are crucial for our application. These are the enhanced direct memory access (EDMA) controller that allows complete data acquisition without central processor unit (CPU) intervention, the multi-unit processor core, which allows up to 8000 million instructions per second (MIPs) to be executed, the glue-less external memory interface (EMIFA and EMIFB) which is capable of interfacing to asynchronous peripherals, such as ADC’s, DAC’s and USB interface chips, and finally the DSK daughtercard interface connectors, which allow additional custom boards to be attached to the DSK. The C6416 DSP incorporates three 32-bit clock/timers, two of which are used to provide clocking support to the ADC daughtercard, with the remaining timer being used to generate precise timing delays. The C6416 DSK has a complete set of development tools which allow rapid application design, including the full featured Code Composer Studio Integrated Development Environment (CSS-IDE) which incorporates an efficient optimizing C/C++ compiler, assembler, linker, debugger, and flexible project manager. The included DSP/BIOS real-time kernel manages real-time and operating system tasks such as boot up, interrupt handling, timer functions, EDMA setup and control, and EMIF initialization. Included with the CCS-IDE is the FlashBurn utility to program code and data into the on-board Flash memory on the C6416 DSK. This allows the user to create a boot loader so that the DSP controller will boot and run in a standalone mode when it is powered up, running independently of the development environment. USB communications are used to connect the development environment to the DSK for program upload, debugging/single stepping, memory viewing, and DSK diagnostics, as well as other tasks.

##### 2.1.2.1 USB/QDAC Daughtercard

**Fig. 5:**
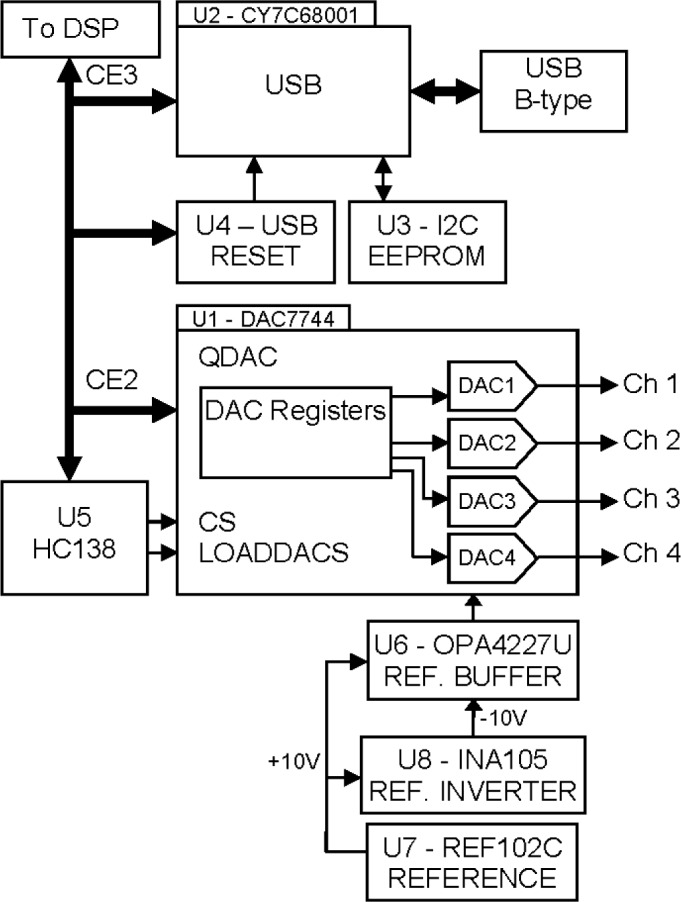
Schematic of USB/QDAC daughtercard showing USB circuitry, daughtercard connectors, quad DAC circuitry and ±10V DAC reference.

The USB/QDAC daughter board incorporates a Cypress CY7C68001 High-Speed USB 2.0 Interface chip capable of operating at high speed (480 Mbps) or full speed (12 Mbps) USB data transfer rates to a USB host. This chip is connected to the DSP via the daughtercard connectors over a 16-bit data bus. Refer to the diagram shown in Fig. 5.

The USB interface consists of the CY7C68001, U2, an M24C01 1 k-bit Serial I2C EEPROM, U3, and a TI TPS3825-33 Supervisory Reset Circuit, U4, which can be connected to the DSP’s reset circuitry to allow the USB to come out of reset shortly after the DSP comes out of reset. U2 connects to the DSP’s EMIFA CE3 memory space, which is completely separate from the memory space, EMIFA CE2, used by the QDAC. There is also a provision for an I2C Serial EEPROM, U3, to be connected to the USB interface and allow it to self-enumerate, but this capability is not used here, i.e., the USB enumerates only under the programmed control of the DSP.

The QDAC circuitry is built around a TI DAC7744 16-Bit Quad Voltage Output DAC, U1. A TI SN74AHC138D 3-line to 8-line decoder/de-multiplexer, U5, is used for address decoding as well as simultaneous-update-register loading via the LOADDACS pin on the DAC7744. LOADDACS allows all four channels of the QDAC to change at the same time, under software control. A TI REF102C low-drift, precision 10V reference, U7, is used as the +10V QDAC reference, and this voltage is inverted by U8, a TI INA105 precision unity gain differential amplifier, to provide a −10V QDAC reference voltage. These two reference voltages are buffered and filtered by U6, a TI OPA4227U high precision, low noise operational amplifier, and applied to the various reference inputs of U1.

Settling time for the DAC7744 is specified to be typically less than 10 µs to within 0.003 % for a 20 V, full scale (−10 V to +10 V) step change. We are unlikely to ever allow the DSP to execute even a fraction of such a full scale step (due to mirror resonance considerations) and so our update rate can likely be even faster than 10 µs, if necessary. Two channels of the QDAC are used to control the position of the analog mirror. This leaves 2 additional DAC channels free to the user for software-programmable tasks. There are many possible uses for these spare channels, such as providing synchronizing signals to allow viewing the mirror scan lines on an oscilloscope, or re-creating any of the 6 signals digitized by the ADC for troubleshooting purposes.

##### 2.1.2.2 ADC Daughtercard

The ADC daughtercard is a TI ADS8365M-EVM evaluation module (EVM) featuring the ADS8365, a 16 bit, 250 kHz, 6 channel, simultaneous-sampling ADC with built-in reference [11].

The EVM uses a TI INA159 low noise difference amplifier on one channel, which allows ±10V signals to be digitized. This is the channel where the image acquisition signal is connected. The second channel can be configured to also accept ±10V signals. This auxiliary channel can be used with the first to acquire two signals at once, such as real and imaginary voltages from a lock-in amplifier, or configured by the user for another purpose. The remaining 4 channels are designed to accept 0 to 5 V signals, which corresponds to what the four optical position sensors integrated into the mirror/gimbal assembly are designed to provide.

The ADS8365 is fully specified at simultaneous sample rates of 100 kHz and 250 kHz for all six channels; these are set by configuring one of the DSP’s timers to generate a sampling clock of 100 kHz or 250 kHz while another timer generates a bit clock of approximately 2 MHz or 5 MHz, respectively. The falling edge of the sampling clock initiates an ADC conversion. After 16.5 bit clock cycles the ADS8365 has completed the conversion of the 1st 2 channels, after 17.5 bit clock cycles, the 2nd 2 channels, and after 18.5 bit clock cycles, the last 2 channels are done. The ADS8365 generates an end-of-conversion (EOC) pulse after each conversion is complete; this EOC pulse is connected to one of the DSP’s external interrupt pins in order to generate an interrupt to notify the DSP. The DSP software responds to the interrupt by reading the data simultaneously from all six channels.

It was determined after significant troubleshooting, that the ADS8365M-EVM had a design flaw which prevents it from functioning correctly in any situation where any other peripheral, such as our QDAC or USB chip, is connected to the DSP data bus. The as-shipped design clearly was tested only in a stand-alone configuration. The solution was to connect a pull-down resistor to the digital output of an auxiliary chip (U7 on the ADS8365M-EVM schematic) which was buffering the chip-select (CS) pin to the ADC.

### 2.2 Software Components

The MLBS software is composed of two components: a bootable, real-time firmware component residing in the DSP’s on-board Flash memory, and a user software component (GUI) which runs on the host computer. These components are connected via a USB-based message-response system which is initiated and controlled by the host. The host is responsible for sending commands and receiving data from the DSP. For example, a typical command could set the number of data points in a scan line, the step size per pixel, or the sample rate for the ADC. Received data includes all DSP parameters and current scan line data.

#### 2.2.1 DSP Firmware

The DSP’s real-time control firmware is responsible for the following:

- generation and control of the scanning algorithm of the MEMS mirror, including static pointing if requested,
- coordination of image data acquisition with mirror scanning,
- applying feedback to control the position of the mirror, if closed-loop position control is activated,
- communication with the user host program.

##### 2.2.1.1 Scanning Algorithm

The scan algorithm includes the choice of a scan type and scan mode parameters. Scans typically have a linear, constant rate of motion section where data is acquired, called the scan line, one or more turnarounds, where the scan reverses direction, and a flyback interval to move to the next scan line, where no data are acquired. Scan types can be raster (2 turn-arounds plus 1 flyback per scan line), bidirectional (1 turn-around per scan line but no flyback), or even spiral (one long, continuous scan line with either 1 turnaround or 1 flyback at the end). Note that, upon reaching the end of a scan line, the DSP must generate a smooth, continuous turn-around in order to avoid exciting resonant modes in the MEMS mirror. We overshoot the defined end of the scan line, using a sinusoidal turn-around waveform which is pre-calculated when the scan algorithm is chosen.

The scan mode parameters select how the scan is calculated and carried out. If the user chooses 100 pixels per scan line and the spacing between pixels is significant, the step movement could cause a resonance in the mirror. We use an oversampling technique to generate finer positioning steps than the user requests in order to avoid mirror problems, e.g., using 10 DAC steps to move between adjacent pixels. This also has the added benefit of allowing a finer resolution for the user to choose where in the scan to acquire image data, in order to accommodate delays associated with time constants in the image acquisition hardware. The time base used for scanning is set by the ADC sample rate, which we set to 100 kHz. This guarantees that the mirror scanning and image acquisition are synchronized to a precise, crystal controlled clock.

Drive current applied to the mirror coils causes the mirror to rotate linearly in angle, so the native units for mirror positioning are angular units. However, the user typically wants to scan a specific sample surface to create an image, so we must convert the native mirror rotation angle into x and y dimensions. This requires that we accurately know the distance from the mirror surface to the sample surface. This distance must be provided by the user, and is used as a global constant to convert angles to x and y coordinates. In addition, the angular rotation per milliamp of drive current is different for each mirror axis, and indeed varies significantly from mirror to mirror. Therefore an accurate calibration constant is required for each mirror axis. This is accomplished by reflecting a laser pointer, whose beam is perpendicular to the surface of the mirror, onto a distant flat surface where a ruler is mounted. The mirror drive current is adjusted from −20 mA to +20 mA. The calibration is determined from the distance moved per milliampere of current and the distance from the mirror to the distant flat surface.

##### 2.2.1.2 Data Acquisition

Data acquisition of the images is accomplished by recording the 16-bit image values and storing them into a linear scan line array. Two scan line arrays, called “ping” and “pong,” are used to temporarily hold the image data. Two arrays are used in order that one may be copied into the DSP’s main memory while the other one is being filled with new image data. DSP main memory, arranged as a two-dimensional array of 16-bit data values, can hold an entire image, up to 16 megabytes. The acquired scan lines are also loaded into the USB output buffer in order to be sent to the host.

The data acquisition is synchronized with the mirror scanning, which is accomplished at a finer resolution than the pixel resolution. For example, if the user chooses a scan line with 100 pixels, but wishes 10 times finer resolution to smooth out the scan, the DSP will create an oversampled scan with 1000 DAC points, but record an image point at only 100 of them. The number of oversamples is set by the user. There is also a provision to allow the user to choose how long after the DAC has programmed a given image pixel before the ADC records the image data. For example, if the appropriate data acquisition delay required is 120 µs after the previous pixel position was reached, and the ADC sample rate is 100 kHz (10 µs), then we record the image data point 12 samples after the previous pixel position. Obviously, this requires that the oversample amount is equal to or greater than 12.

An additional software-activated feature of this system is to require the ADC to record image points for all the DAC oversample points. This allows the user to look at the raw image data and determine exactly when it has settled to the appropriate level. Since this generates a lot of image data, it is generally used only for determining settling time, and then deactivated.

##### 2.2.1.3 Position Feedback

If position feedback is required, the scan/data acquisition algorithm is remarkably similar to the open-loop case. There is still oversampling between pixels, except that instead of having 10 fixed DAC steps per pixel, as in the example above, the number of steps varies by how quickly the mirror responds to the pixel step input. The user can still choose how long to wait after the previous pixel to acquire image data, but the feedback must iterate at least this many times. When feedback control is selected, the ADC sample rate is set to 250 kHz, in order to provide faster closed-loop position settling time. This reduces the performance of the ADC slightly, but since the position feedback circuitry on the TALP1000B is only specified to 13 bits, and the ADC’s 250 kHz performance is better than 14 bits, it should still be acceptable.

A simple proportional-integral (PI) controller is used to provide the feedback control for the mirror. The user sets the PI parameters to provide the fastest mirror response while preventing overshoot or oscillation. Since feedback is active, and the oversampling amount is no longer fixed, the algorithm uses the pixel positions as setpoints for closed-loop positioning. For example, if the user chooses 100 pixels per scan line, then the scan line consists of 100 pixel position setpoints, which take a variable number of DAC steps to execute. Each pixel setpoint is activated, and the feedback is allowed to complete to within a user-defined error margin. During the execution of the following pixel iteration, after the appropriate acquisition delay has elapsed, the image data point for the previous pixel is recorded. If the feedback takes too many iterations to complete, an error condition is generated.

Note that when position feedback is selected, there is no sinusoidal turn-around waveform or special flyback considerations, with the exception of allowing more feedback iterations for a full raster scan flyback to settle than that for a normal single-pixel movement.

##### 2.2.1.4 Host Communication

As indicated above, the scan algorithm is timed by the ADC sample rate, which is an interrupt-driven process that requires the absolute highest priority. Communication with the host is at a lower priority, and runs whenever the interrupt-driven process is inactive. Given the 8000 MIPS processing power and speed of the C6416 DSP, the user should not experience any problems receiving scan lines at the rates allowed by the DSP firmware. However, the scan line number is always sent with the scan line, so that the host program can alert the user if something goes wrong, for example, if a scan line is missed.

##### 2.2.1.5 Overall DSP Algorithm

To understand the overall algorithm running on the DSP we must first consider the timing of the ADC. The DSP clock timer, Timer0, which is fed to the ADC sampling input, is set to run at 100 kHz, unless feedback control is selected, then the sample rate is set to 250 kHz. If the sample rate is 100 kHz, the DSP clock timer, Timer1, which is fed to the ADC clock input, is set to run at approximately 2 MHz, otherwise it is set to approximately 5 MHz. The ADC EOC pin is connected to external DSP interrupt EINT7. The function which services this interrupt is responsible for most of the scanning and acquisition algorithm. If the sample rate is set to 100 kHz, this function executes continuously, every 10 µs (4 µs if the rate is 250 kHz), and performs the following^2^:

Interrupt section: occurs with highest priority at 100 kHz or 250 kHz rate
  - Read the 16-bit data values from all six ADC channels; remember to increment counter responsible for keeping track of data acquisition delay always, even after scan has finished but last data point remains.
  - If feedback is active, calculate the current x and y mirror positions using equations 1a and 1b, compare to the current x and y mirror setpoints and then use this information along with the PI parameters to calculate the feedback correction to the mirror DAC’s. If a turn-around or flyback is called for, allow the feedback extra time, i.e., do not generate an error, to complete the operation. If the feedback has completed to the appropriate error margin and the minimum data delay iterations have completed, then load the next pixel setpoint so that the next feedback cycle will begin to slew to the next pixel position. If the required data delay iterations have elapsed after the previous pixel, record the acquired 16-bit data value for the pixel into the scan line array.
  - If feedback is not active, increment the current x and y mirror positions by the appropriate oversampling pixel interval. If a turn-around or flyback is called for, deactivate the normal scanning increment (while still keeping track of the last pixel delay required and actually recording the last pixel when called for) until the turn-around or flyback has been completed. If the required data delay iterations have elapsed after the previous pixel, record the acquired 16-bit data value for the pixel into the scan line array.
  - If a scan line has been completed, change current pixel storage to the opposite memory array location, i.e., ping or pong.
  - Finally, write the current feedback correction or pixel increments to the x and y mirror DAC’s.
  - Exit interrupt section.
Background section: occurs during the time when the interrupt is inactive
  - Copy appropriate scan lines to DSP main memory.
  - Read commands from host via USB. Act on commands, i.e., update variables (scan parameters, etc.) to reflect user’s wishes. Some commands require immediate action, such as requiring the scan to stop, while others update parameters on the fly, without interrupting the current scan, such as changing the PID parameters.
  - Write host data (completed scan lines and changed scan parameters) to the USB interface.

#### 2.2.2 Graphical User Interface

**Fig. 6:**
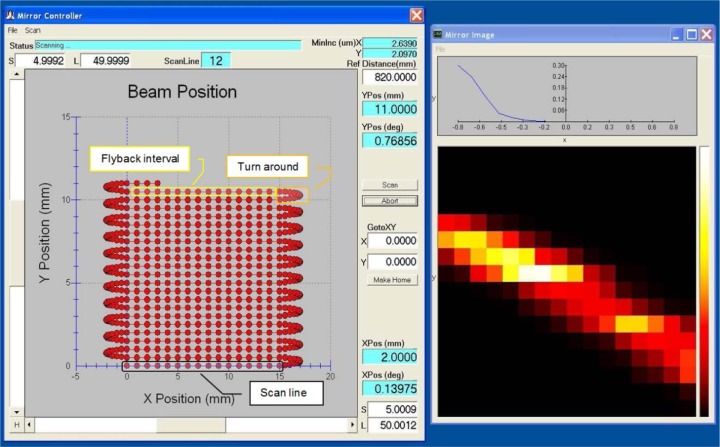
Screen capture of MLBS software GUI showing Mirror Controller panel and Mirror Image display.

The MLBS GUI forms the user control panel for all aspects of mirror scanning and data acquisition. Refer to Fig. 6. As shown in the figure, the GUI consists of one mirror control panel and one image display panel.

The mirror control panel sets or displays:

- User-input x and y coarse and fine step size, for static mirror positioning.
- Current scan line index number: from 1 to the user-input number of scan lines.
- Current x and y position readouts, both in distance units and angular units.
- Current x and y position, shown as a red dot on the position graph; if a scan is in progress, the scan image points, turn-arounds and flybacks are shown in real-time.
- Current x and y minimum increment resolution, for informational purposes.
- User-input reference distance, from mirror to sample surface.
- General static mirror positioning controls: Scroll, Step, Home, Go to.
- Scan control: Start, Abort.
- Status readout: Scanning, Ready, Error, etc.

The File pull-down menu includes:

- Open: Opens a file incorporating saved scan settings.
- Save As: Saves a file with current scan settings.
- Exit: Exits the GUI.

The Scan pull-down menu includes:

- Number of scan lines, which is also equal to the number of image pixels (images are square).
- Pixel step size, for scanning.
- Pixel oversampling.
- Data acquisition post-pixel delay.
- Scan type pull-down: raster, bidirectional.
- Feedback: ON, OFF, PI parameters, error margin, maximum number of iterations.

The mirror image display panel shows the results of the image acquisition. There is a raster display showing the XY pixel plot with color grading. A thermal grading scale (created by linearly ramping red, then green, then blue) is used, and this scale is displayed on the right side of the image display for reference. Above the image display is a graph of the most recent scan line data, i.e. data point voltage vs. pixel index. A File pull-down menu allows current data to be saved or previous data to be displayed.

The GUI was developed using Microsoft Visual BASIC 6.0 (MS VB6). Note that any programming language could have been used with the MLBS as long as it is capable of calling Microsoft Windows dynamic link libraries (DLL) and is capable of displaying graphics and user controls. Note also that the VB6 development environment is *not* a real-time development environment, nor is MS Windows a real-time operating system (OS). The OS for the C6416 DSP, which is named DSP/BIOS by TI, is a real-time OS and is suitable for the tasks described above.

The GUI host communicates with the DSP over the USB interface by sending a short string of commands and data, typically about 4 to 16 bytes. However, the DSP sends much more data to the host; as much as 2K bytes of data can be sent for each scan line. If the host gets interrupted by the Windows OS for some reason, and a scan line is missed, the host can request this missed scan line as soon as it realizes it is missing and the DSP will quickly comply.

## 3. System Performance

### 3.1 Scan Precision and Speed

Scan precision was tested by reflecting the beam from a diode laser off the scanning mirror at an incident angle of 45 degrees and onto a surface a large distance (15 m) away from the mirror. This technique magnifies any noise, distortion, drift, or oscillation that occurs in the mirror. We set the DSP to move the mirror slowly back and forth between two positions, with a continuously decreasing distance between them, until we could no longer see the movement at all. This level, the angular resolution of the mirror movement, was found to be about 0.001 degrees. While doing this measurement, we also checked for any drift in the first position, which was not changed and thus used as a reference position. This drift was found to be about 0.002 degrees over a period of about an hour.

Scan speed, for a 2 degree scan width, was continuously increased until the reflected beam, without feedback, began to show signs of oscillation. This speed, which was found to be about 20 scan lines per second, should never be exceeded in normal operation, in order to prevent measurement errors or damage to the mirror. The scan speed was increased by decreasing the number of scan points from about 10000 points per scan line (2 degrees in 100 ms) to up to 100 points per scan line (2 degrees in 1 ms). This increased the angular deflection rate per pixel by 100X.

As a demonstration of the high-speed scanning capability of the mirror, an optical technique was used, whereby the system was operated essentially in reverse, i.e. no data was acquired via our ADC but instead data was sent to one of our DAC channels.

A small laser diode, similar to that used in a laser pointer was reflected off the mirror surface and onto a flat black screen. The black screen was used to avoid any excess light from reflecting into the aperture of our digital camera, which we used to acquire the image. One of the two spare DAC channels was used to modulate the intensity of the laser diode. The Lenna standard image [12] was used to create the modulation. This image was reduced to a 200 pixel by 200 pixel format with only grayscale information, which left the image with only 8-bits (256 levels) of dynamic range to modulate the laser diode.

In order to draw an image fast enough to be conveniently captured by a conventional camera, we scanned the mirror at a rate much higher than the recommended rate of about 20 scan lines per second. Though we don’t recommend operating this way in general, it can be done for this specific case, in which we stayed far away from any resonance. The resonant frequency of the gimbal/mirror is specified to be 152 Hz maximum; therefore we scanned the mirror above its resonance, at 200 scan lines per second, in its x-axis. The y-axis scans were fine enough at 200 lines per image that no special considerations were necessary and we just stepped the y-axis the appropriate distance per x-axis scan line to give a square image. No retrace was done; the image was simply scanned continuously up and down.

A pure sinusoid was used for the scanning at an amplitude sufficient to give an image on our screen of about 120 mm width and height when the mirror was approximately 3 m away from the screen. The sinusoid was created with 500 points at 100 kHz, and the 200 points centered around the positive slope were modulated with pixel information. This section is not completely linear, but it did suffice to produce a high quality image. The laser was dimmed but not turned off during the remaining 300 points. The amplitude of the laser modulation was adjusted during the acquisition of the image in order to give the best contrast. Since the scan rate is 200 lines per second a complete image frame is created every second.

**Fig. 7:**
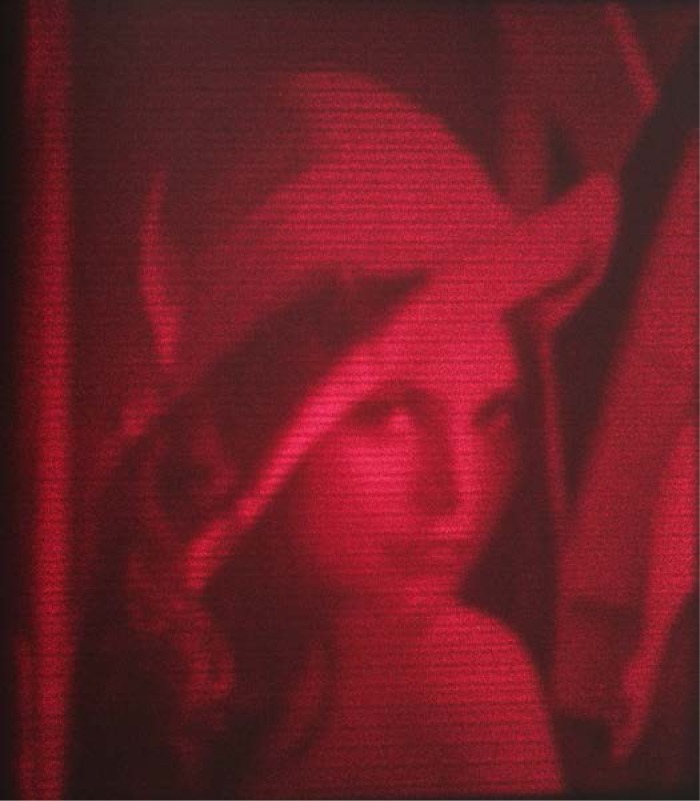
MLBS scanned image captured with a digital camera.

The image captured with our camera is shown in Fig. 7. The camera was set to an 8 second exposure (8 frames exactly) in darkness, using a tripod and delay on the shutter trip to avoid vibration. Some banding is apparent in the image but the clarity is good, given that the image was created in one second at a 200 scan line per second rate, with 10 microsecond pixels. We believe this represents the ultimate speed performance of our mirror system. However, for use as a beam chopper, which is a much simpler operation, with constant frequency and sufficient amplitude, we have successfully operated the mirror at as high as 1 kHz scan rates. A powerful feature of this system is that this can all be done with simple changes in software.

The MLBS unit was also mounted into a 850 GHz (352 µm) beam path. The mirror was steered ±2 degrees and the beams were characterized for distortions. No distortions were observed. A more careful mapping of the beams’ shapes will be performed and published in future papers.

### 3.2 ADC Noise

The ADC noise level, as manifested in the completed system, with enclosure covers in place, was tested by terminating the measurement input BNC connector in 50 Ω and recording data for several complete scans, then calculating the rms value and standard deviation in this data. Several scan increment levels and arbitrary data delays were used. The results indicate that the ADC noise level is about 2 mV rms. This is equivalent to about 7 bits.

## 4. Summary and Future Work

We demonstrated a new practical approach for steering long-wavelength beams by use of MEMS-mirror technology. Our MLBS unit employs a fast DSP controller in order to allow the user to produce a scanned image of a sample with undistorted and high spatial-resolution (many thousands of pixels). Alternatively, a significantly faster (line scans in seconds) scanned image can be produced but with only a few hundreds of pixels. Imaging systems are designed in such a way to achieve large area coverage with a coarse resolution. Once interesting smaller regions are identified, the imager is switched to its high resolution mode in order to uncover smaller size features on the sample. The MLBS characteristics allows for such dual operation without any modification to the imaging system. Furthermore, the high speed and precision of the MEMS-mirror pointing will replace mechanical choppers due to its higher switching speeds and the substantial reduction in the overall physical dimensions of the unit.

As terahertz/optical imaging and spectroscopic systems are introduced to a wide range of applications and the requirement for portability, high resolution, and sensitivity become paramount, the dimensions of the scanning elements of the systems must be significantly reduced but with improved operation. Currently, imaging systems with very large number of pixels require complicated and costly multiplexing schemes. While such multiplexing schemes are cumbersome for systems based on room-temperature detector technologies, the complexity increases several fold when cryogenic detector based systems are needed for the highest sensitivity applications. Our MLBS introduces an alternative multiplexing approach at the front-end of terahertz systems.

Finally, both terahertz imaging and spectroscopic systems can be combined for identification and diagnostics of biological and chemical agents present on an image of a sample. A number of characteristic frequencies can be sampled by tuning the local oscillator and/or designing an array of terahertz detector elements that detect these different characteristic frequencies. The MLBS is capable of operating in multiple frequencies adjusting for the pixel size and resolution by merely changing a few parameters in the control program. We are planning on introducing this technology to a number of terahertz systems currently under development.
